# Integrating the Perspectives of Youth, Women, and Marginalised Communities in Addressing Global Environmental Management Challenges

**DOI:** 10.1007/s00267-025-02254-7

**Published:** 2025-08-28

**Authors:** Richard Kwame Adom, Gbenga Abayomi Afuye, Ahmed Mukalazi Kalumba, Mulala Danny Simatele

**Affiliations:** 1 School of Geography, Archaeology and Environmental Studies, University of Witwatersrand, Johannesburg, South Africa; 2 Department of Geography and Environmental Science, University of Fort Hare, Private Bag X1314, Alice, Eastern Cape Province South Africa; 3 Geospatial Application, Climate Change and Environmental Sustainability Lab–GACCES, University of Fort Hare, Alice, Eastern Cape Province South Africa

**Keywords:** Community Empowerment, Environment and Youth, Equitable Solutions, Gender and Sustainability, Population Dynamics

## Abstract

Globally, environmental mismanagement, overconsumption, population growth, and lifestyle changes disproportionately impact society, particularly affecting marginalized and vulnerable groups in developing nations. Despite progress in raising awareness and funding, many initiatives, including Youth Engagement Programs, Capacity Building, Gender (women) Mainstreaming, and Community-Based Approaches, have been limited or ineffective due to demographic variability. This study employed qualitative and quantitative methods to examine institutional factors that hinder the effective use of demographic variables in addressing global environmental challenges. It explores how institutional and structural factors obstruct the integration of demographic variables into global environmental policies and programs. The results show that over 65 percent of the global population, particularly youth, women, and marginalized groups, remain passive victims of environmental disasters and are largely excluded from national and international decision-making platforms. This study reveals significant gender disparities in environmental knowledge, interest, and decision-making across Sub-Saharan Africa, the Middle East, North Africa, and parts of Asia, largely shaped by cultural traditions and norms. This study advocates for targeted capacity-building initiatives and the integration of indigenous knowledge to foster inclusive growth, enhance knowledge-sharing mechanisms, and address the underrepresentation of youth, women, and marginalized groups. These findings offer evidence-based insights for policymakers, researchers, and environmental organizations to enhance global environmental governance by promoting inclusivity and innovative solutions that empower these groups for active participation in policy decision-making.

## Introduction

The 21st century is grappling with significant environmental challenges such as a scarcity of clean freshwater, ecosystem degradation, soil erosion, biodiversity loss, atmospheric chemistry changes, and climate change, necessitating sustainable solutions (McSweeney and Coomes, 2020; Afuye et al., 2022). The implications of these environmental hazards are felt disproportionately across different geographical locations and population divides (Afuye et al., 2021a; Khine et al., 2023). Bhargara and Bhargara (2023) assert that environmental degradation impacts society’s fabric, particularly the youth, women, and marginalized groups, particularly in global south countries, due to their economic vulnerability, resource scarcity, high dependency on natural resources, and intergenerational inequity. Marginalized groups in this context refer to communities or population that are socially, economically, and politically disadvantaged and, as a result, have limited access to environmental decision-making, resources and benefits (Adley et al., 2024). Despite being worst affected by environmental damages, these groups’ involvement in environmental issues is often limited or restricted (Scheidel et al., 2020). Addressing environmental challenges requires integrating perspectives from all sectors, including demographic variables and marginalized groups in policy formulation and implementation, as their disadvantage is widely acknowledged (Alexopoulos et al., 2023; Berrone et al., 2023). Hariram et al. (2023) emphasize the importance of demographic variables in understanding the intricate relationship between human populations and environmental management, thereby promoting global environmental sustainability. Hariram et al. (2023) argue that these factors significantly influence how communities interact with environmental policies and respond to sustainability initiatives. According to Hariram et al. (2023), the analysis of these variables will force policymakers to design targeted interventions that account for population-specific needs and behaviours, ultimately improving the effectiveness of environmental management strategies. (Karuga et al., 2023). Climate change and other environmental challenges are exacerbated by the exclusion of the marginalized populations from decision-making due to geographical vulnerability, historical injustices, undervaluation of traditional knowledge, and political exclusion (Amorim-Maia et al., 2022). These marginalized populations are relevant to promote inclusivity, equity, justice, culturally appropriate solutions, community resilience, policy effectiveness, environmental justice advocacy, and capacity building for proactive environmental issue management (Tanner et al., 2022; Prendergast et al. 2021).

Over the past five decades, international institutions and interest groups such as the United Nations Environmental Programmes (UNEP), Youth Climate Movement (YCM), Women’s Environment and Development Organisation (WEDO), and Indigenous Peoples’ Organisation (IPO) have integrated marginalized groups into decision-making programs on environmental issues, primarily in developed and advanced countries. (Sibiya et al., 2022). Canada’s Feminist International Assistance Policy, United Nations Women’s Empowerment Principles, Women’s Environment and Development Organisation, and the Global Gender Office of the International Union for Conservation of Nature are among the organizations promoting women’s participation in environmental issues (Caswell and Jang, 2024). Others include Gender Equality for Climate Change Opportunities, Community-Based Natural Resource Management, Women Entrepreneurs for Renewable Energy and Gender Action Plans in Climate Change Policy (Bryan et al., 2024, Adom et al., 2022). Programs such as Indigenous Peoples’ and Local Communities’ Conserved Areas and Territories aim to integrate marginalized groups, such as traditional leaders, Indigenous people, the poor, and less educated population in addressing global environmental challenges (Dawson et al., 2021). The inclusion of youth, gender, and marginalized groups’ perspectives in global environmental management policies and legislation is being made significant by agencies and institutions in Sweden, Canada, Costa Rica, Norway, New Zealand, Rwanda, and Kenya (Badgett et al., 2019). Most countries, especially in the global south, Eastern Europe, and South Asia, have not achieved the desired goals of integrating these population groups into environmental management (Kwauk et al., 2023). The authors argue that most African and third-world countries are grappling with a gap between well-developed policies and inadequate implementation.

The exclusion of youth, women, and marginalized groups from decision-making is a result of a multifaceted issue involving underrepresentation, structural barriers, socioeconomic inequalities, and cultural norms (James et al., 2023). Tanner et al. (2022) identified systemic and institutional barriers, such as insufficient empowerment, lack of transformative action, limited political will, weak capacity, and regulatory capture, as impediments to these groups’ participation. Deininger et al. (2023) highlighted that women worldwide are significantly underrepresented in decision-making on critical issues at local and national levels. Erikson et al. (2020) found that women make up less than 25 percent of national parliamentarians globally, and they hold only 12 percent of top ministerial positions in environment-related departments at international, national, regional, district, and community-level committees. Mavisakalyan et al. (2020) emphasised the significant impact of the lack of women in global decision-making on environmental management. This includes issues such as limited perspectives, gender bias, missed innovation opportunities, unequal resource distribution, slow women’s empowerment progress, and weak resilience and adaptation efforts that are prevalent. Youth and other marginalized populations face similar scenarios in environmental management. Nsor and Alhassan (2015) and Verkooijen (2021) reported that the global population aged 15–24 is currently 1.2 billion, representing 16 percent, of the world’s population and is expected to reach 1.34 billion by 2050. The evidence indicates that these population groups are not actively involved in global environmental management programs (Piscitelli and D’ Uggento, 2022). Salvatore and Wolbring (2021) argues that excluding marginalized groups such as youth from global environmental management is a significant setback. Limitations and underrepresentation hinder decision-making, underutilize talent, innovation, creativity, and untapped Indigenous knowledge, and unfairly distribute environmental worth (Sibiya et al., 2022; Weber and Sciubba, 2019). Karo (2018) highlighted the lack of autonomous organization, weak coordination, and clear direction regarding the roles and responsibilities of marginalized groups in global environmental management. The lack of follow-up for environmental management activities and the absence of civic societies are key factors contributing to passiveness and non-participation in these activities.

Studies have highlighted the importance of incorporating the perspectives of youth, gender, and marginalized groups in addressing global environmental management challenges (Chang et al., 2022; Muttarak, 2021). The exclusion of critical groups in global environmental management is attributed to factors such as limited access to education, resources, socioeconomic disparities, discrimination, cultural norms, lack of representation, capacity building, political and institutional barriers, geographical and socio-cultural isolation, lack of recognition of traditional knowledge, and lack of funding (Kuran et al., 2020; Adom et al., 2024b). Similarly, Karo (2018) highlights that there is a lack of autonomous organisational structure, weak coordination, and a lack of clear-cut direction regarding the responsibilities of youth, gender and those marginalised in environmental management. Furthermore, the absence of follow-up on environmental management activities, the nonexistence of civic societies to mobilise them into unit force nationally and internationally have fundamentally undermined their contribution to environmental issues globally.

Considering these constraints, several scholars, policymakers and commentators, including Karo (2018), Muttarak (2021), Eastwood et al. (2017) and Simatele et al. (2018), explored these issues and identified weak coordination among various environmental groups, poor channels of communication, lack of commitment and a lack of prioritising environmental issues among the population, and proposed different models and strategies to address global environmental challenges. However, the current literature lacks a comprehensive understanding of power dynamics and the impact of demographic variables on environmental management and decision-making processes. Considering these gaps, this study delves into the underlying factors contributing to the exclusion of youth, gender, and marginalized groups from global environmental management under these three main objectives:

- To explore the relationship between socioeconomic demographic factors on environmental decision-making.

- To assess the effectiveness of the current integration programs involving women, those marginalized, and youth’s perspectives in addressing global environmental issues and the institutional and structural factors hindering these programs, and
- To identify alternative strategies for empowering youth, those marginalized and women’s participation in environmental issues.

- This paper thus seeks to enhance research by addressing knowledge gaps in inclusive environmental governance, advancing intersectional climate action, and providing empirical insights into the barriers and opportunities faced by youth, women, and marginalized communities. It seeks to inform policy by advocating for equitable frameworks, inclusive decision-making, and mainstreaming participation in climate governance. Practically, it seeks to strengthens community resilience, build leadership capacity, and improve the implementation of global environmental agreements by ensuring the active involvement of underrepresented groups in sustainability efforts. The rest of the paper is structured as follows:
- The introduction provides an overview of the research problem, objectives, and significance.
- The literature review examines existing studies on the integration of youth, gender, and marginalized groups in global environmental management.
- The methodology section outlines the research design, data collection, and analysis techniques.
- The results and discussion present key findings, highlighting challenges and opportunities for inclusive environmental governance.
- Finally, the conclusion summarizes the main insights, policy implications, and recommendations for future research and practice.

## Literature Review

### Demographic Variables and their Influence on Global Environmental Management

The Earth’s population has grown from six billion in 2010 to over eight billion currently (Gu et al., 2021). Global child mortality rates have decreased by over 60 percent, life expectancy has increased by more than 20 years, people are living healthier, are better nourished, indicating positive development and contributing to a healthier world (Sadigov, 2022). The Millennium Ecosystem Assessment report highlights global environmental degradation, alarming global warming, increased pollution, resource depletion, and rising sea levels as significant threats to human survival (Cork et al., 2019; Afuye et al., 2021b). The universal understanding is that population growth has contributed significantly to environmental challenges (Gamelon et al., 2019). Muttarak (2021) emphasized the importance of demographic factors such as population composition, distribution, and social and economic status in shaping societal dynamics. Factors such as income, education, ethnicity, cultural practices, and technological advancements significantly influence environmental changes and decision-making processes at local, national, regional, and international levels (Malapane et al., 2024).

Population variations and social and economic status significantly influence the environment and its management, as different subgroups exhibit varying behaviours and interests towards its management (O’Sullivan, 2021). Milan-Garcia and Caparros-Martinez (2021) highlight that the global population currently comprises the largest group of young people aged twenty-four and below, the largest proportion of the elderly, and the highest levels of migration in human history. The influx of a largely younger generation into urban towns is expected to increase urbanization and intensify environmental challenges, posing significant challenges to the environment and society (Ostby, 2016). Scholars such as Sharma et al. (2018) and Ivanova et al. (2016) argue that rapidly growing populations in developing regions are the main cause of environmental damage, while Wiedmann et al. (2020) and Stuart et al. (2020) focus on the environmental costs of overconsumption among developed countries. Sharma et al. (2018) discovered that the wealthiest quarter of the world’s population consumes over three-quarters of natural resources and contribute to 70 percent of global waste, highlighting the disproportionate impact of these issues. Stuart et al. (2020) found that the United States, Germany, Japan, and China collectively produce over half of the world’s economic output, while over 500 million people in Sub-Saharan Africa share the same amount as 10 million in Belgium. Excessive energy and natural resource use in industrialized countries lead to pollution, global warming, climate change, and over nutrition, causing diseases and death among their population (Milan-Garcia and Caparros-Martinez, 2021). At the same time, poverty in many developing countries leads to deforestation, damage to watersheds, and land degradation (Burki et al., 2021). The industrialized nations must balance their high consumption and productivity by shifting investment, research, development, productive capacity, management, and other skills to the developing world (Greenford et al., 2020).

Developed countries must enhance efficiency by minimizing waste and paying full prices for goods and raw materials imported from developing nations (Montt and Harsdorff, 2018). Education, traditional knowledge, and cultural and ethnic backgrounds are intrinsically linked to these factors. Erhabor and Don (2016) found that education significantly influences environmental decision-making, as higher levels of education lead to increased environmental awareness and knowledge, resulting in more informed decisions. Individuals who are educated are more likely to understand environmental issues, their consequences, and potential solutions, leading to increased engagement in sustainable practices and support for environmental policies (Mohiuddin et al., 2018). Cultural and ethnic backgrounds significantly influence environmental changes, with ethnic groups shaping environmental decision-making through various mechanisms (Medina et al., 2019). The authors suggest that cultural values, beliefs, and environmental experiences significantly influence attitudes and behaviours, and Indigenous knowledge is crucial in environmental management, especially in Africa and other developing countries (Medina et al., 2019). Sultana and Muhammad (2018) highlighted the integration of indigenous knowledge in sustainable practices, biodiversity conservation, climate change adaptation, natural resource management, cultural heritage preservation, community resilience, medicinal plants, and ecological wisdom. Collective wisdom, deeply rooted in local ecosystems and cultural traditions, provides valuable insights for sustainable development and promoting harmonious human-environment relationships (Sultana and Muhammad, 2018). Burgo-Ayala et al. (2020) reveal that combining indigenous knowledge with modern scientific methods can enhance the effectiveness and cultural sensitivity of environmental management practices globally. In summary, it is clear from the literature that demographic factors and the socio-economic status of the population influences the global environmental management. These population groups shape policy effectiveness, public participation, resource access, and adaptive capacity. These factors determine how different populations engage with sustainability initiatives, respond to environmental policies, and adopt eco-friendly practices. They also affect decision-making processes, resilience to climate challenges, and the distribution of environmental resources. Therefore, neglecting these variables in policy design can lead to ineffective interventions and limited success in achieving sustainability goals. Therefore, integrating demographic considerations into environmental management is essential for fostering inclusive and impactful sustainability strategies.

### Global Environment Policies, Institutional and Structural Challenges to Implementation

- Since the 1960s, the United Nations Environmental Programme (UNEP) has provided global frameworks for improving environmental management (Purvis et al., 2018). Different international policies have been formulated based on their commitment, and international conventions have been signed to manage the global environment and use resources sustainably for overall development (Escobar-Pemberthy and Ivannova, 2020; Mensah and Cascidevall, 2019). Similarly, Purvis et al. (2018) highlighted that these global institutions aimed to create environmental awareness and facilitate international cooperation that addresses global environmental challenges such as climate change, global warming, deforestation, and biodiversity loss. Some of these polices and strategies include:
- The 1972 Stockholm Declaration on Human Environment the 1987 Montreal Protocol banning the use of Chlorofluorocarbons (CFCs).

- The 1997 Kyoto Protocol seeking to reduce greenhouse emissions and combat climate change.
- The United Nations Framework Convention on Climate Change (UNFCCC).
- The Paris Agreement and both COP 26 and 27.
- The Conference on Environment and Earth Summit which drove world attention on sustainability and natural resources, and set out in Agenda 21 a plan of action for future global partnerships; biodiversity loss through the Convention on Biological Diversity (CBD), the land degradation through the United Nations Convention to Combat Desertification (UNCCD), the protection of endangered species through the Convention on International Trade in Endangered Species on Wild Fauna and Flora (CITES), as well as the United Nations Convention on the Conservation of Migratory Species of Wild Animals (Gupta et al., 2022; Mitchells, 2019, Karlsson-Vinkhuyzen et al., 2022 and Escobar-Pemberthy and Ivannova, 2020; Afuye et al., 2024).

Notably, these conventions are incorporated into Sustainable Development Goals (SDGs), which emphasise achieving international sustainability ambitions, preserving essential ecological and biological diversity, encouraging sustainable exploitation of natural resources, improving environmental relations, and enhancing understanding of the linkages between environment and development (Osborn et al., 2015).

**Fig. 1:**
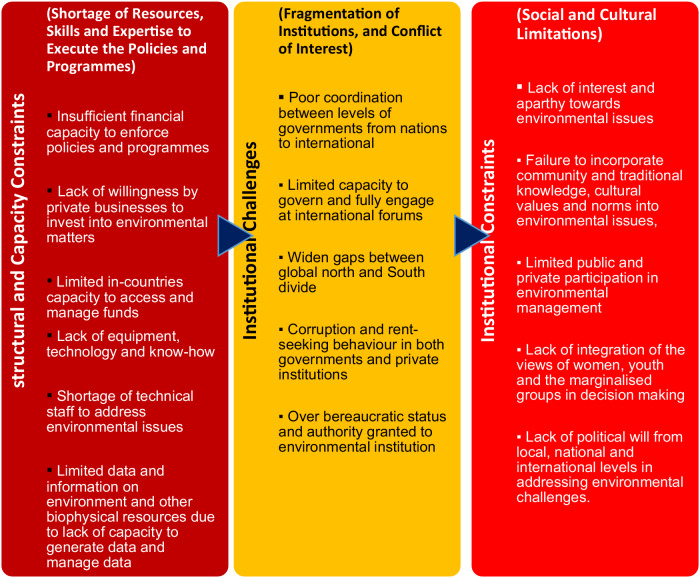
Structural, institutional, social, and cultural impediments to youth, women, and marginalised participation in environmental issues. **Source:** United Nations Magazine 2020

Many scholars and commentators, such as Howes et al. (2017), Carattini et al. (2020), and Hazemba and Halog (2021), acknowledged that there have been some achievements, such as the marked rise in cooperation among the 166 member states of UNEP to protect and preserve the global environment in the last few decades. Nonetheless, numerous scholars, such as Karlsson-Vinkhuyzen et al., (2022) and Roos et al. (2020), concurred that widespread structural and institutional constraints hinder the enforcement of the regulations and policies to combat global environmental threats. Howes et al. (2017) identified the structural constraints as being economic, social, political, technical, legal, administrative and communication failures. Similarly, Cerna (2013) opined that the lack of political support for environmental goals, the ineffectiveness of environmental institutions (particularly about implementation and enforcement), the inability of governments to mobilise funding to tackle environmental issues, weak environmental policy integration and the failure to establish monitoring progress and set new targets, remained significant at global levels. Le Quesne et al. (2010) posited that the lack of youth, gender, and marginalised population participation in global environmental management is intricately shaped by a combination of structural, institutional, social, and cultural hindrances. Figure 1 uses the flow of information arrows to illustrate the interconnectedness of three categories, indicating that each constraint related to environmental policies and programs in one section can exacerbate another, creating a complex network of challenges. The hierarchical, but interrelated challenges depicted, highlight the multifaceted nature of obstacles to address environmental matters, distinguishing different layers of issues. The structural and capacity constraints report highlights the lack of resources, skills, and expertise required for the effective implementation of environmental policies and programs. The institutional constraints category addresses the fragmentation of institutions and conflicts of interest that hinder effective environmental governance. The social and cultural limitations discuss the lack of interest and apathy towards environmental issues, highlighting cultural and societal challenges (Fig. 1).

The linkages between institutional, structural, social, and cultural hindrances to youth, gender, and marginalised groups’ involvement in environmental management are interconnected and mutually reinforcing (Deininger et al., 2023). Structural constraints, such as economic disparities and lack of infrastructure influence institutional limitations on capacity and resource allocation (Goga and Mondliwa, 2021). The institutional challenges further shape social and cultural challenges through policy and decision-making processes, perpetuating exclusionary practices (Bopape, 2021). The social and cultural constraints become entrenched structural hindrances by influencing resource allocation and societal norms (Grindstaff, 2022). Galsanjigmed and Sekiguchi (2023) further posited that institutional barriers exacerbate social and cultural hindrances by reinforcing stereotypes and discrimination, while social and cultural hindrances further perpetuate institutional barriers by influencing decision-making processes and practices. These authors believe that understanding these interconnections is essential for developing comprehensive strategies that address barriers to inclusive participation in environmental management (Fig. 1).

### Theoretical Framework of Youth and Gender Participation in Environmental Issues

Many scholars, environmentalists, and policymakers, including Thew (2018), Salvatore and Wolbring (2021) and Shiratuddin et al. (2021), are of the view that the lack of meaningful participation by the youth and gender-diverse individuals in global environmental issues can be attributed to complexities and interplay theories. Ahmed et al. (2021) identified some of these theories as being structural inequality, intersectionality, neoliberalism, feminist political ecology, youth engagement, critical political economy, social capital, and cultural and social norms.

**Fig. 2:**
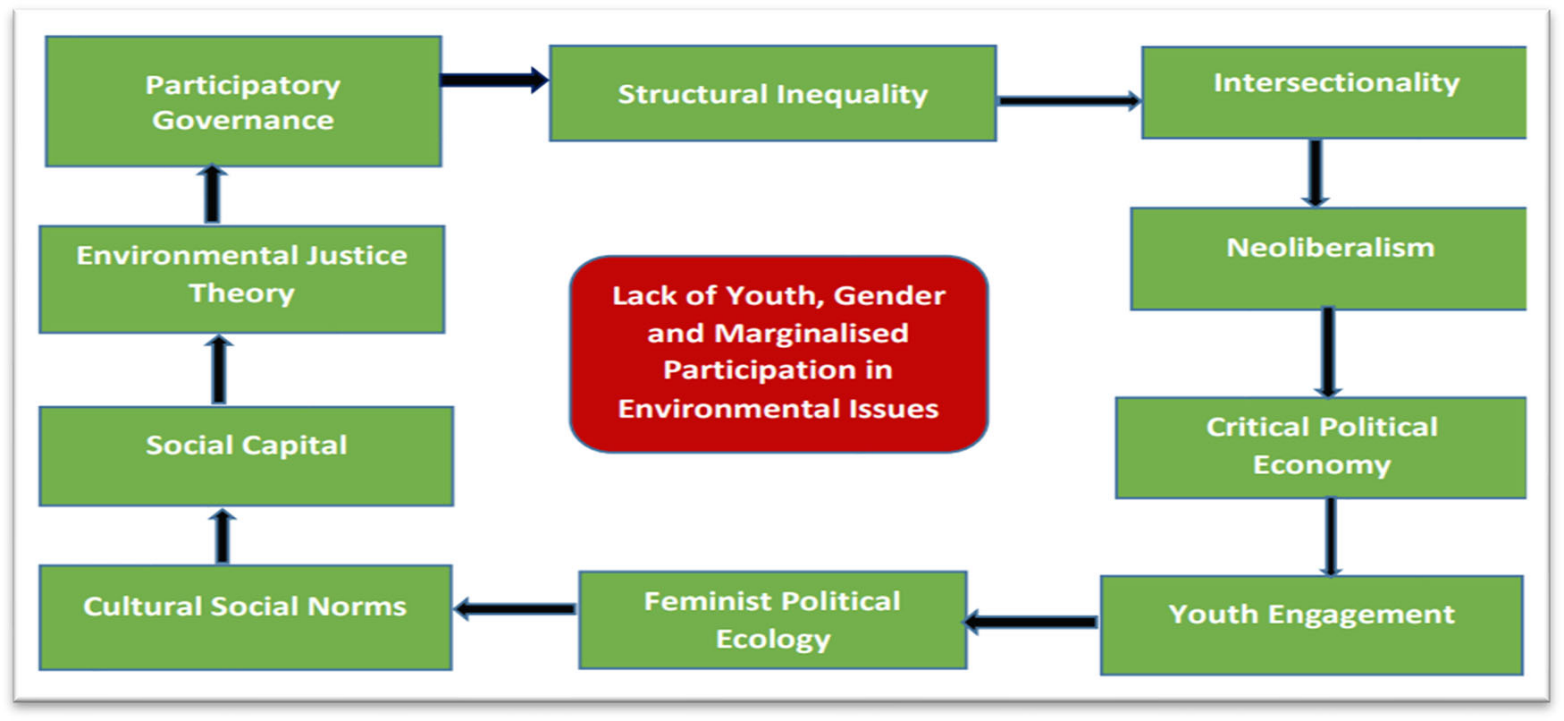
The theoretical framework of youth, gender marginalised participation in the environment

For this study, the Structural Inequality Theory is employed to analyse how systemic social, economic, and political disparities contribute to the underrepresentation of youth, marginalized communities, and women in global environmental management. Structural barriers, including limited access to decision-making platforms, unequal resource distribution, and socio-economic constraints, perpetuate exclusion from critical environmental governance processes (Tucker et al., 2023; Kalumba et al., 2025). Despite global commitments to inclusive climate action, power dynamics and entrenched institutional biases continue to side-line these groups, limiting their ability to influence policy and adaptation strategies. This study explores how these inequalities hinder meaningful participation and examines potential pathways to foster more inclusive environmental governance (Sibiya et al., 2023). Prager et al. (2015) argue that structural disparities perpetuate inequalities in access to education, resources, and decision-making among age and gender groups on matters dealing with the environment. These inequalities are exacerbated by the intersection of identities and other factors expounded by an intersectionality theory. Butler (2021) argues that investing resources in the youth on environmental issues is a long-term goal that falls outside the interests of corporations and other organisations. Baycan et al. (2023) stated that cultural and social norms reinforce exclusionary practices, while limited social capital further hinders marginalised groups’ participation. Skinner-Thompson (2022) expressed that environmental justice frameworks underscore the disproportionate burden on marginalised communities, while participatory governance strategies fail to address underlying power imbalances in environmental management. King et al. (2021) are of the view that collectively, these factors create multifaceted barriers to the youth, women, and other marginalised groups from active participation in environmental issues at local, national and international stages. Addressing these challenges requires a comprehensive strategy that challenges power dynamics, dismantles systemic inequalities, and prioritises the voices and needs of marginalised groups in decision-making. Figure 2 presents a structural inequality framework that emphasises the lack of youth and gender-marginalized participation in environmental management, illustrating interconnected concepts and potential pathways.

The analysis focuses on the lack of youth, gender, and marginalized participation in environmental issues, highlighting their underrepresentation in governance and decision-making processes (Fig. 2). The central issue (red box) analysis highlights the underrepresentation of youth, gender, and marginalized individuals in environmental issues, highlighting their lack of involvement in governance and decision-making processes. The surrounding (green boxes) represent concepts or theories that are influenced by the central issue, with arrows indicating the direction of the influence. Fundamentally, this theory advocates that the lack of youth, women and marginalized involvement in global environmental management is the result of interconnected social, political, and economic structures that create systemic exclusion. Primarily, cultural social norms limit access to social capital, which in turn weakens the influence of environmental justice theory that drives and shapes inclusive participatory governance. This governance gap reinforces structural inequality, which acts as a bottleneck, preventing marginalized voices from engaging in environmental decision-making. On the economic and political front, structural inequality intersects with neoliberalism and a critical political economy that prioritize market-driven solutions over community-based resilience strategies. This intersection exacerbates exclusion, making youth engagement more difficult. Simultaneously, the framework of feminist political ecology challenges these inequities but is often undermined by entrenched socio-political barriers. The combined effect of these forces perpetuates the exclusion of marginalized groups, reinforcing a cycle where they remain absent from critical environmental governance and policy decisions. The advocators of this theory such as Elmhirst (2022), Gonzalez et al. (2023) and Chipango (2022), highlight that addressing these directional effects requires structural reforms that integrate intersectional approaches, participatory governance, and equitable resource distribution that ensure inclusive environmental decision-making.

## Materials and Methods

### Research design

Addressing global environmental challenges requires broader engagements with various stakeholders in environmental management institutions and organisations. Considering these, this study employed a concurrent mixed research of qualitative and quantitative approaches to explore the institutional and structural hindrances affecting the youth, women and the marginalised groups participating in global environmental issues. A mixed research approach, according to Shorten and Smith (2017), is a research method that combines and integrates qualitative and quantitative research methods in a single research study. It involves collecting and analysing qualitative and quantitative data to understand a phenomenon better and answer the research questions. The qualitative approach was employed to understand the perspectives of youth, gender, and marginalized groups on environmental issues, identify factors limiting their participation, and devise strategies to enhance their involvement in global environmental issues. The quantitative method was used to ascertain the demographic and socioeconomic status of the respondents. To assess the demographic and socioeconomic status of respondents, this study utilised the composite index approach that incorporates various demographic and socioeconomic factors (Poirier et al., 2020; Adom et al., 2024a). The framework for calculating the demographic and socioeconomic status index is given by Eq. (1)

1 $${DSSI}=\mathop{\sum }\limits_{i=1}^{n}{W}_{i}{\times F}_{i}$$

where, ${DSSI}$ is the Demographic and Socioeconomic Status Index, ${W}_{i}$ is the weight assigned to each demographic or socioeconomic factor $i$, based on its relative importance, ${F}_{i}$ is the score or value of each factor $i$ for a respondent and $n$ is the number of factors considered (age, gender, country’s categorisation, education, employment status, etc.). The (DSSI) was calculated using a weighted additive method. Relevant variables (such as age, gender, education, employment) were selected, scored (standardized or ranked), and assigned weights based on expert input, literature, or statistical methods like PCA. Each respondent’s index score was computed by multiplying each factor’s score (F_i_) by its weight (W_i_) and summing across all factors. The final index was normalized (0–1 or 0–100) for comparability. Robustness checks included sensitivity analysis and internal consistency testing.

### Sample Population and Sampling Techniques

This study utilized purposive and random sampling techniques to collect data. Purposive sampling is an intentional selection of informants based on their ability to elucidate a specific theme, concept, and phenomenon (Campbell et al., 2010). Researchers selected 12 respondents from United Nations Environmental Programme (UNEP) secretariats from six continents. Participants were selected from Nairobi in Kenya, Pretoria in South Africa from the African region, Geneva in Switzerland, Brussels in Belgium, and Berlin in Germany from the European region. Others included Beijing in China, New Delhi in India from the Asian continent, Kingston in Jamaica, Panama City in Panama, Rio de Janeiro in Brazil from the Caribbean and Latin America, and New York in the United States of America for the North American region. These respondents were chosen purposely to obtain holistic perspectives of global environmental challenges. The respondents were deliberately chosen based on their positions in the various secretariats, the experiences they possessed and their involvement in either the formulation or implementation of policies and strategies for global environmental programmes. The interviews were conducted strictly by telephone, Zoom meetings, and Google meeting platforms between March and June 2022. These virtual techniques were used mainly due to COVID-19 restrictions at the time when human contact was prohibited.

The quantitative data was obtained using a random sampling method. Random sampling is the randomised selection of a small segment of individuals or members from a whole population (Igwe et al., 2020). This approach applied various methods to invite potential participants to take part in the survey. Firstly, participants were selected through random sampling by sending out emails to youth, women, and marginalized groups via invitations and shared links on social media. Additionally, participants were randomly recruited through relevant environmental management networks and organizations. This study recognized potential selection bias, as participants were largely drawn from environmental networks, resulting in higher levels of knowledge and engagement than the general population. This may have led to an overestimation of environmental awareness, concern, and advocacy in the findings. To mitigate this bias, the study included individuals from non-environmental backgrounds, rural communities, and less-engaged groups, ensuring a more representative and unbiased dataset for analysis. Additionally, data from environmental groups were compared with broader population surveys to assess potential bias and derive a more balanced perspective on environmental engagement.

Secondly, language barriers were addressed to ensure inclusivity among respondents from diverse regions and continents. Primarily, questionnaires in English were designed; however, an integrating translation link ensured that local language challenges and cultural sensitivity issues were addressed. Similarly, permission notes from parents and institutions were obtained from respondents under the age of 18. The questionnaires assisted in identifying the demographic backgrounds of the respondents and their levels of understanding of environmental management. Both the quantitative and qualitative data were carried out simultaneously between March and June 2022. Based on the large data obtained, a sampling error of 5 percent was calculated on the data collected. Our sampling method relied on voluntary participation, which may have introduced biases and affected the external validity of our results. Therefore, the equation for the margin of error $(E)$ in proportion is estimated as:

2 $$E=Z\,\times \sqrt{\frac{P\,(1\,-\,P)\,}{n}}$$

where, $E$ is the margin of error (or sampling error), $Z$ is the Z-score corresponding to the desired confidence level (e.g., for 95% confidence, $Z$ is approximately 1.96), $p$ is the estimated population proportion (a decimal between 0 and 1), and $n$ is the sample size. The margin of error formula was used to estimate the level of uncertainty in the proportion results obtained from our sample. By inputting the estimated population proportion (P), the sample size (n), and the Z-score corresponding to a 95% confidence level (typically 1.96), we calculated the range within which the true population proportion is likely to fall. This helped assess the reliability of the findings despite the non-random nature of the sample.

### Data Collection Method

The study utilized primary questionnaires, interviews, and secondary data to examine demographic perspectives on global environmental challenges. A cumulative number of 5000 responses were received from the online survey of youth between 15–35 years of age, women, and the marginalised population from different countries across six continents. Specifically, 605 responses were received from Africa (South Africa, Kenya, Ghana), 612 from Asia (India, China, and Singapore), 800 from North America (United States of America, Canada, and Costa Rica), 735 from Latin America (Brazil, Colombia, and Ecuador), 680 from Australia (Australia, New Zealand, and Papua New Guinea) and 1568 responses were received from Europe (Britain, Switzerland, Belgium, and Germany). The questionnaires were used to gather data on demographic variables such as age, gender, educational levels, country, and region of residence, annual and standard of living, employment status, knowledge, awareness, and passion for environmental issues. Table 1 summarised the total number of respondents.

**Table 1:** Demographic Information of Respondents

Region	Countries Included	Number of Responses	Age Range	Men	Women	Others
**Africa**	South Africa, Kenya, Ghana	605	15–35	400	150	55
**Asia**	India, China, Singapore	612	15–35	410	145	57
**North America**	United States, Canada, Costa Rica	800	15–35	520	200	80
**Latin America**	Brazil, Colombia, Ecuador	735	15–35	485	180	70
**Australia**	Australia, New Zealand, Papua New Guinea	680	15–35	450	170	60
**Europe**	Britain, Switzerland, Belgium, Germany	1568	15–35	1044	366	158
**Total**	**-**	**5000**		**3309**	**1211**	**480**

**Source:** Field survey, 2022

In terms of qualitative data, a total of 12 online interviews were conducted with directors and administrators of UNEP secretariats in 12 countries. The interviews focused on various environmental policies and programmes within the United Nations, as well as structural and institutional barriers to integrating the youth, gender and marginalised perspectives into global environmental programmes and alternative strategies to enhance demographic participation in the decision-making of ecological programmes. The secondary data in the form of reports, policy documents, journals, book chapters, and other information were used to complement the primary data obtained and the policies guiding global environmental management activities.

### Data Analysis

The responses obtained from the questionnaires and the interviews were analysed concurrently using different analysis techniques. The quantitative data obtained was analysed using Statistical Package for the Social Sciences (SPSS) version 30 software. This method facilitated data capture and analysis, producing frequency tables and graphs for quick interpretation, understanding, and reading of the gathered data. The study analysed qualitative data on demographic variation, structural, systemic, and institutional constraints to youth, gender, and marginalized participation in global environmental issues, and strategies to enhance demographic participation using thematic analytical techniques. Thematic analysis is a method for qualitative data that entails searching across a data set to identify, analyse and report repeated patterns (Kiger and Varpio, 2020). This analytical technique was used to classify data into themes for interpretation and discussion.

## Empirical Evidence and Analysis

The data obtained from the survey were organised and structured based on the objectives set up for the study, which included the perceptions of the population towards environmental issues, structural and institutional factors hindering youth and women participation and strategies for improving youth and gender participation in solution orientation research.

### The Perceptions of Social Factors on Youth and Gender on Environmental Issues

This paper explored the relationship between the social and economic backgrounds of the respondents. These variables were used to gauge the population’s perceptions, attitudes, and interests towards environmental issues. Table 1 reflects the demographic backgrounds of the respondents.

The respondents incorporated into the survey were those between the ages of 15 to 35, as shown in Table 1. According to the United Nations demographic data of 2012, these age cohorts are regarded as youth. The survey evaluated environmental awareness among the respondents using socioeconomic and demographic factors, including gender, age, education, geography, employment, and economic development of the country. These variables were used to establish a holistic picture of global environmental issues; out of 5000 responses, 3200 represented 64 percent males, and 1400 (28%) were females. Regarding their educational backgrounds, most respondents, 41 percent, had a bachelor’s degree, while 22 percent possessed postgraduate qualifications; only 4 percent of the study population had no formal education. Most of the population, 41 percent, reside in cities, against 21 percent who dwell in rural communities; of the surveyed group, 47 percent were single, and 29 percent were either students or fully employed. Most of the responses, 1200 (48%), were received from developed countries, compared to 32 percent and 20 percent from emerging and less developed countries, respectively. This statistical breakdown was validated by some respondents who shared their views in interviews: A climate change activist from Geneva, Switzerland, said this:

“I am a 21-year-old environmental activist from a developed country; age means different things in our contemporary world; it can be either a barrier or an advantage in terms of environmental activism. As a young person, I always bring fresh perspectives and innovative ideas to the table. However, older generations often dismiss my/our concerns or underestimate our capabilities. Additionally, young people often lack the resources and experience to influence decision-making processes effectively”.

Another climate change activist from Panama City in Panama in the Caribbean expressed this in an interview:

“I am a 28-year-old female environmental scientist from a developing country; in my view, gender plays a significant role in shaping the participation of youth in environmental management. In many cultures, traditional gender roles limit girls’ access to education and opportunities for leadership. Even in more advanced countries, women are often underrepresented in STEM fields and environmental policymaking. As a female scientist, I’ve faced discrimination and scepticism in male-dominated spaces”.

A climate change activist from a marginalised community in Brazil stated this in an interview:

“Marginalisation exacerbates the challenges youth and women face in participating in global environmental management. Indigenous peoples and marginalised communities are disproportionately affected by environmental degradation and climate change, yet their voices are often silenced or ignored. Structural inequalities, land dispossession, and lack of access to resources perpetuate environmental injustice. As an Indigenous activist, I’m fighting for the recognition of Indigenous rights, traditional Knowledge, and community-based solutions to environmental problems”.

These views highlight the complex interplay between demographic characteristics and youth participation in global environmental management, underscoring the need for inclusive approaches that address participation barriers and amplify marginalised communities’ voices.

### The Involvement, Interest and Knowledge of Youth, Women and Marginalised in Global Environmental Management Issues

**Fig. 3:**
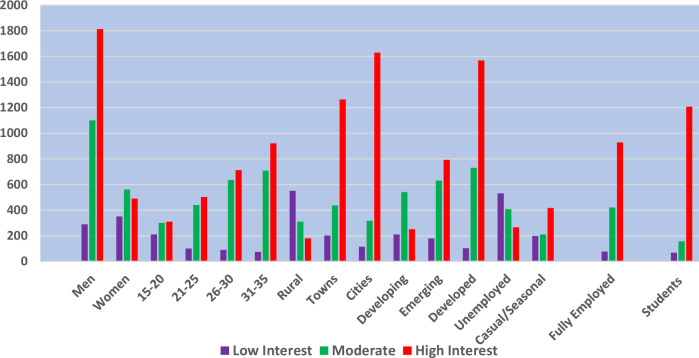
Respondents’ involvement, interests, and knowledge of environmental matters. **Source:** Field survey, 2022

An assessment was carried out to measure the study population’s perception, knowledge, and interest regarding the environment using stated variables. Respondents were asked these questions: “What level of knowledge do you have about global environmental management issues; how strong is your interest in these issues; and in what specific ways have you been actively involved in addressing environmental challenges at the local, national or global level?” Fig. 3 shows a bar chart illustrating respondents’ involvement, interests, and knowledge of environmental matters across various demographics and social categories, with a range of values from 0 to 2000 representing the number of respondents.

High interest was notably observed in men, city dwellers, emerging economies, employed individuals, and students, while moderate interest was more balanced across men, younger age groups, and rural areas, and low interest remained low except for some younger age groups. Consequently, the breakdown of the data obtained revealed that men were more interested, more involved, and more informed about environmental issues than women. This manifested in the answers obtained. Among the 5000 responses collected, 1812 men, representing 36 percent, expressed a high level of interest in environmental issues. In contrast, only 490 women, or 10 percent, showed a similarly high level of interest. Additionally, 1100 men reported a moderate interest, compared to 560 women. Conversely, 288 men demonstrated a low level of interest, compared to 350 women with low interest in environmental issues. Other variables indicated that 73 percent of urban and city residents rated their interest in environmental issues as high or moderate, compared to 27 percent of rural inhabitants. On average, 70 percent of respondents who are educated, urban, employed, and from developed countries are more informed, interested, and knowledgeable about environmental issues, whereas only 30 percent of those who are less educated, rural, unemployed, and from less developed countries demonstrated similar levels of engagement. Online interviews with directors of UNEP in Brussels, Beijing reaffirmed the statistical data breakdown in Fig. 1. Their message was that:

“Many young people across the globe, particularly those from third world countries, are apathetic towards environmental issues because they perceive that their personal views or actions won’t make any difference. This has often led to demotivation and indifference to global environmental issues.”

Other interviewees from New Delhi, India and Pretoria, South Africa, stated that:

“Many youth and marginalised people, particularly from developing countries, are constrained from participating in environmental issues due to social and economic circumstances. These groups face socioeconomic constraints that limit their ability to engage in global environmental issues. Their immediate concerns are survival needs such as food, jobs, shelter, and clothes”.

Overall, Fig. 3 shows that demographic factors such as women, older age groups (26–35), city dwellers, those in emerging economies, and students tended to show higher levels of involvement and knowledge of environmental issues. The high interest in these categories indicates that these groups are likely to be more proactive in environmental matters.

### Levels of Involvement and Commitments to Environmental Activities

The paper further assessed the levels of involvement by youth, women, and the marginalised in undertaking environmental activities, such as environmental advocacy and lobbying, community clean-up campaigns, tree planting campaigns, or environmental education programs. Respondents were asked to rate how often they get involved in addressing environmental challenges in their communities and beyond. The responses we received showed a significant majority of 3756 of 5000, 75 percent, do not participate in any environmental programmes; this is followed by 850, representing 17 percent, who stated that they scarcely participate in environmental activities. Only 322 respondents, equating to 6 percent, responded that they frequently engaged in environmental activities. This statistical breakdown is validated by an interview with a Director of UNEP who is based in Nairobi, Kenya. She stated that:

“Many of the global youth, women, and marginalised people, particularly those from less developed economies, are not participating in environmental activities mainly because no comprehensive environmental activities target these population groups. Even though there has been growing awareness, the resources to support these initiatives are completely missing”.

This view was backed by another Director of UNEP representative based in Kingston, Jamaica. His concern was that:

“Young people have lost faith in global institutions, particularly the UNEP system of solving global environmental problems. These bodies are seen as institutions of good policies and ideas rather than good executions of results. He continued that youth and women’s participation is not being harnessed effectively since member states have failed to support communication with the understanding of the youth”

A director from New York America who contributed to the issue expounded during an interview that:

“Many young people are eager to contribute to environmental issues. Just look at millions of school-age students marching for greater actions from governments to address environmental challenges. However, they don’t have the skills, resources, or political and technical abilities to tackle the challenges. Their main constraints are oppressive social norms such as age and gender inequality, making it difficult for their voices to reach those in power”.

A common view that cuts across our engagement suggests that extensive environmental management awareness creation needs to be enforced to boost the perception of the youth and women in the environment.

### Challenges Hindering Youth, Gender, and Marginalised Participation in Environmental Issues Globally

One of the key objectives driving this research is to uncover the factors hindering youth and women’s participation in global environmental issues. Hence, respondents were asked to categorise a set of listed constraints from lesser to severe obstacles. Respondents’ comments were calculated using the Likert Scale of Range. Using the formula, answers obtained were classified under: Not an obstacle, Less of an obstacle, Major and Very Severe obstacles: 0 ≤ x ≤ 350 = Not an obstacle; 350 ≤ x ≤ 700 = Less of an obstacle; 700 ≤ x ≤ 1500 = Major obstacle and 1500 ≤ x ≤ 5000 = Very severe obstacle. The explanation of this formula is simplified as follows:

- 0 ≤ x ≤ 350: This range indicates that when the value of “x” falls between 0 and 350, it is considered “Not an obstacle”. This suggests that whatever “x” represents, it poses no significant hindrance or challenge within this range.
- 350 ≤ x ≤ 700: When “x” falls within this range, it is classified as “Less of an obstacle”. This implies that there might be some minor challenges or impediments associated with the value of “x” in this range, but they are not major hindrances.
- 700 ≤ x ≤ 1500: In this range, “x” is categorized as a “Major obstacle”. This suggests that the value of “x” in this range represents a significant challenge or hurdle that needs to be addressed or overcome.
- 1500 ≤ x ≤ 5000: When “x” falls within this range, it is labelled as a “Very severe obstacle”. This indicates that the value of “x” in this range represents a substantial and possibly critical barrier or challenge that requires urgent attention or significant resources to address. Table 3 depicts the answers of respondents.

The respondents rated the 12 obstacles assessed as “very severe” and six as “major” constraints to women and youth participation in global environmental issues. Only a small number of the respondents regarded the obstacles as not issues and barriers to the participation of these demographic groups. The director of UNEP representative in Geneva, Switzerland, supported this statistical breakdown. This interviewee stated:

“Decision-making spaces are largely inaccessible to women and young people because institutions and cultural norms are set up for these groups to take orders from the elderly, mostly men, and not to ask questions. The spaces do not consider their challenges, such as lack of resources, the timing of the events, study needs and access to information. More importantly, when engaged, they are reduced to tokenistic or shallow-level engagements deprived of deeper ones. In other words, just a tick-box exercise”.

Similar views were shared by a director from Beijing, China when commenting on this issue. His perspectives revealed that:

“Most young people do not have sufficient knowledge or understanding of environmental issues and their general impact. Inadequate education and limited awareness campaigns hinder their engagement and willingness to participate”.

Another interviewee from the UNEP office in Panama City disclosed during our engagement:

“Numerous educational systems fail to prioritise environmental subjects and fail to adequately empower the young population with knowledge and expertise required to understand and tackle global environmental constraints”.

The common view emanating from most respondents was that women and youth are neglected in key decision-making involving environmental management, though these groups form most of the global demographic distribution.

### Enhancing Youth and Gender-Base Activities in Global Environmental Management

**Fig. 4:**
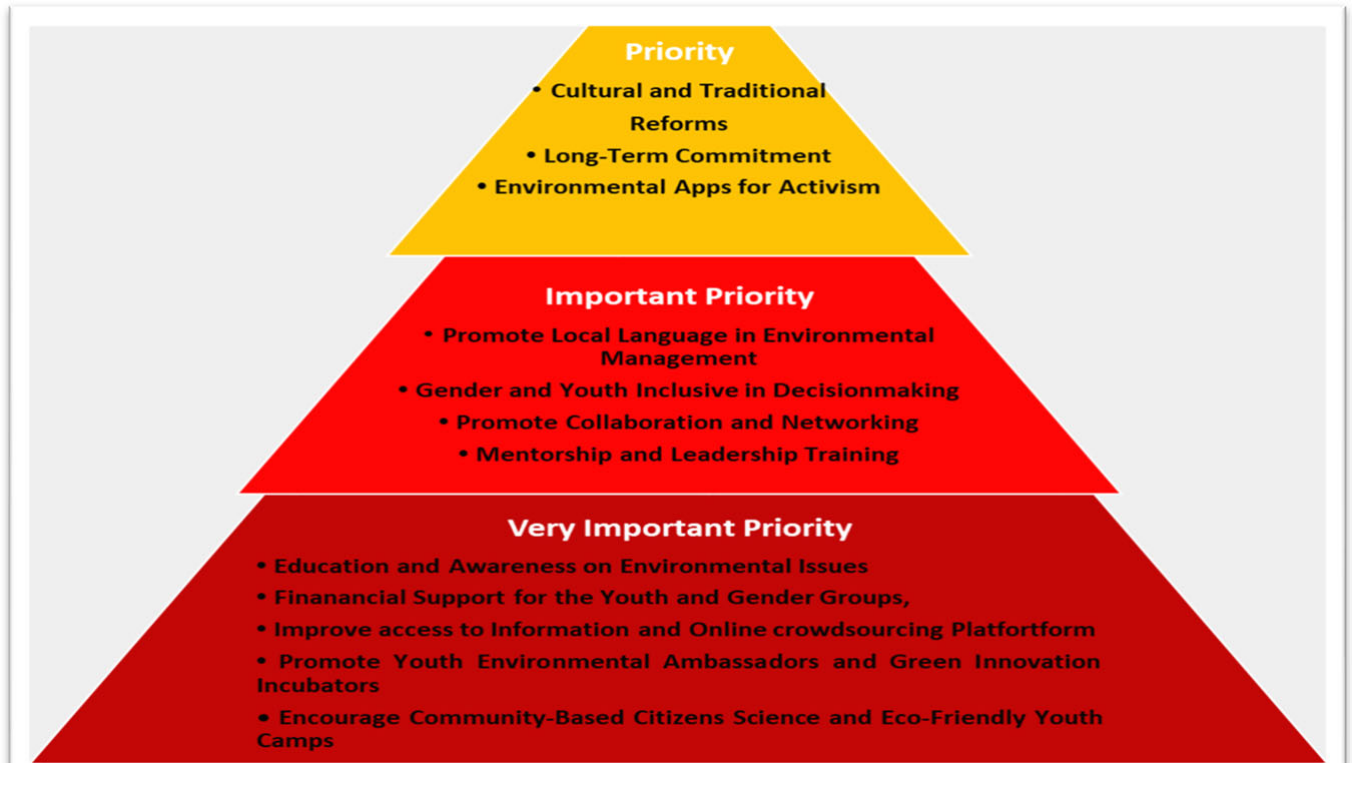
Strategies for enhancing youth and gender participation in environmental activities. **Source:** Field survey, 2022

Considering the structural and entrenched discrimination against the youth and women in environmental management, this study explored strategies for improving these groups’ participation in environmental activities. Respondents were asked to pick their preferred approaches to addressing global challenges from a set of strategies. Using a Likert scale, the researchers calculated and classified respondents’ answers under the following variables: “**Priority, Important Priority and Very Important Priority**”. Under this formula, a normal and ordering approach was used of **(**<**less than; = equal to; > greater than; ≤ less or equal to ≠ not equal to; ≥ greater or equal to and x to equate numbers within a range)** to calculate the answers provided. In this equation, responses were compiled as: **[0** ≤ **x** ≤ **1000 equal to “Priority”; 1000** ≤ **x** ≤ **2500 equate to “Important Priority” and 2500** ≤ **x** ≤ **5000 represent “Very Important Priority”]**. The snapshot of the formula suggests that if the value falls between 0 and 1000 (inclusive), it is categorised as “Priority”. If the value falls between 1000 and 2500 (inclusive), it is classified as “Important Priority”. If the value falls between 2500 and 5000 (inclusive), it is labelled as “Very Important Priority”. Figure 4 shows respondents ‘ responses, illustrating a pyramid-shaped hierarchy of strategies to increase youth and gender participation in environmental activities, and it ranked from the most critical to the least urgent based on their priorities. The Very Important Priority (base of the pyramid) is a strategy to increase youth and gender participation in environmental activities by enhancing essential knowledge, resources, and opportunities for sustainable practices and environmental awareness. The Important Priority (middle of the pyramid) tier focuses on strategies that are important but follow foundational ones, focusing on creating inclusive and supportive environments for youth and gender groups. Priority (top of the pyramid) is less urgent than those in lower sections, focusing on long-term and systemic reforms that could strengthen efforts from lower tiers.

Out of the 12 strategies considered to enhance participation in environmental management, five of them, representing 42 percent, were regarded as important priorities, suggesting that they are urgently required to address these challenges; four of them (33%) were considered as either important or priority strategies that must be instituted to address youth and gender involvement in either local or global environmental management. The statistical information was supported by interviewees who were engaged in interviews. A UN representative in New York stated this during an online discussion:

“Improving youth and gender participation in global environmental issues will require implementing comprehensive environmental education programmes in schools and communities that raise awareness and passion among these population groups. This must be done through workshops, seminars, and online platforms. Moreover, there must be strong collaboration between governments, NGOs, academia, and private sectors to create a coordinated support structure to support youth and gender initiatives and promote research on youth and gender dynamics in various fields of environmental management”.

Another interviewee from the UN agency in Brussels, Belgium, stated:

“To increase youth and gender participation in environmental matters, international organisations such as the United Nations, African Union, European Union, and other regional blocks must establish structures within their institutions purposively for the youth and women on environmental issues with well-funded resources to address environmental challenges. Similarly, they must be active participants in policy formulation and implementation at local, national, and global levels”.

Overall, the pyramid structure suggests a hierarchy of needs, with foundational elements such as education, financial support, and basic information (base) being addressed first. Once these are established, efforts can shift towards systemic changes such as inclusive decision-making, mentorship, and cultural reforms (top). The strategies cover a comprehensive approach to enhancing youth and gender participation in environmental issues, focusing on capacity-building, inclusivity, leadership, and the use of technology. The engagements suggest that incorporating the voices of the youth and gender is crucial in addressing global environmental challenges, as they are often overlooked in current efforts to tackle these issues.

## Discussion

This study is structured to provide a comprehensive understanding of the contributions and challenges of youth, gender, and marginalized populations in global environmental management. This involves examining the relationship between socioeconomic and demographic factors in global environmental decision-making and evaluating the effectiveness of existing integration programs that incorporate the perspectives of women, marginalized groups, and youth in addressing environmental challenges. This study examines the institutional and structural constraints hindering these goals and explores alternative strategies to enhance the participation of youth, marginalized groups, and women in global environmental management.

### Social, Economic and Demographic Influence on Environmental Issues by the Youth, Gender, and Marginalised Groups

This study discusses the relationship between demographic dynamism and environmental decision-making, as well as the obstacles faced by youth and gender in environmental challenges and suggests strategies to enhance their involvement in global environmental issues. The first objective of this paper is to establish the connections between social, economic, and demographic characteristics and the knowledge, interest, perceptions, and awareness of global environmental issues. Findings from Table 2 and views from some participants uncovered that socioeconomic and demographic factors significantly influence the knowledge and interest of the youth, women, and marginalised groups in environmental matters. The underlying outcomes of Table 2 and Fig. 3 suggest that over 70 percent of educated individuals with high and stable incomes and access to information, urban dwellers and generally men, are more informed, involved, and more interested in global environmental issues due to greater access to education, media, digital resources and exposure to environmental policies and discussions compared to rural, poor, rural dwellers and women who often have limited access to formal education, digital media and global policy discussion, resulting in lower awareness and engagement with international environmental concerns.

**Table 2:** Socio-demographic profile of respondents

Demographic Variables	Groupings	Number of Respondents	Percentage of Respondents
**Age**	15–20	624	12.5%
	21–25	1021	20.4%
	26–30	2273	45.5%
	31–35	1082	21.6%
**Categorisation of countries**	Developed	2324	46.5%
	Developing	987	19.7%
	Emerging	1689	33.9%
**Gender**	Male	3309	66.2%
	Female	1211	24.2%
	Others	480	09.6%
**Marital status**	Married	823	16.5%
	Single	2372	47.4%
	Divorced	233	04.6%
	Not stated	1572	31.4%
**Educational background**	No education	161	3.2%
	Primary	257	5.1%
	Secondary	553	11.1%
	Diploma	841	16.8%
	Bachelor	2123	41.5%
	Postgraduate	1065	21.3%
**Geographical location**	Rural	1067	21.3%
	Towns	1906	38.1%
	Cities	2027	40.5%
**Employment status**	Student	1433	28.7%
	Unemployed	1201	24.0%
	Fully employed	1428	28.6%
		824	16.5%
	Seasonally employed	114	02.3%

**Source:** Field survey, 2022

Other convincing and glaring outcomes of Table 2 and Fig. 3 further proved that social, economic, and demographic factors that influence understanding, interest and commitments towards environmental issues are uneven across countries, regions and continents. These findings underscored the fact that the complex interplay of factors such as levels of economic development, resource availability, population density, urbanisation, cultural practices, government priorities, governance effectiveness, access to technology and education, climate vulnerability and historical legacies significantly affect the attitude of the youth, women and marginalised on the environment issues. The findings in Table 2 demonstrate that the majority of the youth, women and marginalised populations from the global north nations, particularly North America, Western Europe, East Asia and Oceania, regions and continents characterised by strong economies, technological innovation, well-developed infrastructure, high levels of education and healthcare and higher standards of living are more abreast, knowledgeable and more interested in global environmental issues compared to similar population groups from the less educated families, lower-income and developing countries mainly from Africa, Middle East, Eastern Europe and South Asia whose economies are weak and vulnerable. They focus more on survival issues and pay little attention to environmental issues. These findings are also shared by Tsey et al. (2021), that population factors such as gender, age, and education, as well as social and economic factors such as per capita income and individual income status, influence their knowledge, interest and activism on environmental issues. Dhange et al. (2022) also shared similar views that while women, the youth, and the marginalised are disproportionately negatively affected by environmental changes, their participation and association with environmental issues are driven by intersection factors, including power dynamics, socioeconomic circumstances and societal norms and expectations. It was further established that environmental activism among the population, particularly the youth, gender, and the disadvantaged, is intrinsically linked to a country’s short to long-term goals. These outcomes aligned well with Fig. 2, the theoretical framework of the study. The framework stresses that the lack of youth, gender, and marginalized participation in global environmental management is deeply rooted in structural inequalities. The framework underscored the intersectionality and overlapping systems of oppression of gender, youth, and marginalised population exclusion due to neoliberalism and critical politics and market-driven policies prioritising economic interests over marginalized voices. Furthermore, feminist political ecology and cultural social norms highlight gendered power dynamics limiting women’s participation, reinforcing systemic barriers. The framework further uncovered that limited social capital precludes youth and marginalized groups from influencing policies, a challenge explained by the environmental justice theory, which calls for more equitable governance structures. While participatory governance and youth engagement mechanisms enhance inclusivity, systemic barriers restrict any meaningful involvement. These findings underscore the fact that due to the availability of resources, most developed and advanced countries have integrated sustainability and long-term benefits of the environment into their national environmental policies and have raised the population’s consciousness towards these values. This contrasts with less developed countries, mainly in the global south, whose limited resources and competing priorities take precedence over environmental sustainability.

### Structural Barriers, Institutional Constraints and Other Challenges to Gender, Youth, and Marginalised Involvement in Environmental Matters

Findings from Table 3 revealed that structural, institutional, and cultural factors collectively limit youth, women, and marginalised populations from participating effectively in global environmental management. The findings underscore the fact that while these common constraints have stalled the youth, women, and marginalised groups from active participation in global environmental issues, nonetheless, the intensity and prevalence of the challenges vary across different countries and continents mainly due to socioeconomic disparities, cultural norms, political contexts, environmental challenges, access to technology and information, legal frameworks, and international dynamics. The underpinning view emanating from Table 3 and engagements from different respondents suggest that the global disparities in accessing resources, technologies, information, and political systems have hindered the involvement of youth, gender, and marginalised groups in the participation of global environmental management in developing countries, unlike developed and advanced countries. It was uncovered that lack of access to quality education, a dearth of technologies and infrastructure development, as well as funding gaps, have entrenched youth apathy, gender inequalities and marginalisation of communities mainly in Sub-Saharan Africa, South Asia, and Indigenous communities in the Amazon in Brazil from environmental activities. Similar findings are also shared by Sanderson et al. (2021), who disclosed that structural and institutional barriers such as limited funding opportunities, absence of organisational knowledge, lack of technical skills, a dearth of motivation, lack of political will and massive information deficit have contributed to the redundancy of youth, women, and the marginalised population from active involvement of environmental management, particularly in the third world countries. Nevertheless, part of the findings uncovered that some of the developed countries, including the United States of America, the United Kingdom, Australia, Canada, and New Zealand, have limited participation of their youth, gender and marginalised population in global environmental issues due to factors such as complacency and disconnect stemming from stable environmental conditions, a technological dependency, political apathy, prioritisation of economic growth over environmental education, and influence of corporate interests on environmental policies. These findings are also shared by Ding et al. (2019) and Minneti et al. (2018) that even in developed countries such as Sweden, USA and Germany, with strong environmental governance frameworks, there are still barriers to effectively integrate youth, gender, and marginalised populations in decision-making involving environmental issues. However, there are some successes where structural reforms have significantly increased the participation of youth, women, and marginalized groups in environmental management across different regions (Mwaura et al., 2021). Conrad (2023) cited Kenya, the Youth Climate Action Network (YCAN), integrating young people into national climate policies, enabling over 5,000 youth to contribute to sustainable initiatives. Whereas in India, the Self-Employed Women’s Association (SEWA) had empowered over two million women through microfinance, training, and policy recognition in climate adaptation (Gracy et al., 2024). Similarly, in Canada, the Indigenous Climate Action Network (ICAN) has secured indigenous representation in global climate governance, leading to stronger land protections and biodiversity conservation (Vogel et al., 2022; Afuye et al., 2025). These cases highlight the importance of institutionalizing inclusivity to enhance climate resilience and sustainability worldwide.

**Table 3:** Constraints to Youth and Women’s Participation in Global Environmental Issues

	Not an Obstacle	Per. %	Less an Obstacle	Per %	Major Obstacle	Per.%	Very Severe Obstacle	Per.%	Total No. Respondent	Total %
Structural and Capacity Barriers										
Limited financial support for the youth, women and the marginalised	300	6%	360	7%	1900	38%	2440	48%	5000	100%
Lack of technical capacity for environmental programmes	170	3%	230	5%	2000	40%	2600	52%	5000	100%
Overlapping sectoral objectives within global programmes	460	9%	900	18%	2100	42%	1540	31%	5000	100%
Limited availability of information for the youth and women	140	3%	244	4%	1500	30%	3136	63%	5000	100%
Time constraints and priorities among global youth and women	210	4%	315	6%	3025	61%	1450	29%	5000	100%
Institutional and Conflict of Interest										
Inadequate institutional coordination among youth programmes	136	3%	480	10%	1280	26%	3344	67%	5000	100%
Lack of autonomous organisational structures	196	4%	440	9%	660	13%	3704	74%	5000	100%
Weak policy and institutional framework globally	230	5%	500	10%	2440	49%	1830	37%	5000	100%
Poor coordination among international, national and local structures	396	10%	1060	21%	1500	30%	2044	41%	5000	100%
Limited/lack of environmental programmes for the youth globally	103	3%	215	4%	543	11%	4139	83%	5000	100%
Social, Economic and Cultural Obstacle										
Lack of youth women representation	100	2%	150	3%	320	6%	4430	89%	5000	100%
Lack of transparency in environmental issues by business organisation	206	4%	286	6%	1440	29%	3068	61%	5000	100%
Lack of field programmes that involve women and youth	230	5%	396	8%	1400	28%	2974	59%	5000	100%
Lack of confidence in youth and women in environmental issues globally	206	4%	440	9%	2759	53%	1600	32%	5000	100%
Poor training and curriculum in schools, colleges and universities	196	4%	214	4%	1630	33%	2960	59%	5000	100%
Communication and language barrier among youth globally	200	4%	318	6%	2884	58%	1598	32%	5000	100%
Skepticism and apathy among women youth and the marginalised	344	6%	453	9%	2765	55%	1438	29%	5000	100%

**Source:** Field survey, 2022

### Improving Youth and Gender Participation in Global Environmental Management

The underlying findings of Fig. 4 suggest that addressing the challenges of exclusion of the youth, gender and the marginalised from global environmental programmes ought to be addressed in phases from short to long-term priorities, taking into consideration national, regional, and continental social, economic, cultural, and environmental circumstances. The primary finding from Fig. 4 revealed that inculcating the youth, gender and marginalised groups into environmental management, particularly in developing and emerging regions such as Africa, South Asia, Eastern Europe and Latin America, will require a comprehensive strategy that encompasses educational awareness, capacity building and empowerment, improving access to funding and information and active citizenry from local to continental levels. These findings are also expressed by the Columbia Global Policy Initiative (CGPI, 2014), which states that improving youth, gender, and marginalised groups’ participation in global environmental management will require structural and functional environmental education and awareness programmes from community to national levels. Furthermore, it was established that it is critical to develop an integrated framework globally where the youth, gender and marginalised views and contributions on environmental issues should be meticulously considered and incorporated into programs of action by United Nations Environmental Programmes, United Nations Development Programmes and United Nations Entity for Gender Equality and the Empowerment of Women and other national and private organisations. These outcomes are similar to the perspectives shared by Shiratuddin et al. (2017) that there is a need to develop a worldwide action plan to increase youth, gender and marginalised participation, particularly in decision-making and implementation processes of global environmental management. These findings are similar to Noor and Fatima’s (2012) theory in environmental management by the youth in Pakistan. These authors advocate for empowering youth, gender and marginalised populations through environmental education and training, fostering community involvement, raising awareness and nurturing partnerships and collaboration, encouraging innovation and creativity, and advocating for supportive policies, all to mobilise these demographic groups to play an active role in addressing environmental challenges and promoting sustainability within their locality, national, regions and internationally.

### Limitation of the Study

Conducting the study on integrating youth, gender, and marginalized groups into global environmental management presented several challenges that influenced the comprehensiveness of the findings and the generalizability of the recommendations. One of the primary limitations was the availability and reliability of data, particularly in the Global South, where gaps in demographic and environmental datasets hindered a more detailed analysis. Additionally, variations in policy frameworks, governance structures, and institutional capacities across countries created inconsistencies, making it difficult to establish universally applicable strategies. Efforts to incorporate diverse perspectives were further constrained by structural barriers such as language differences, digital accessibility issues, and political restrictions, which limited the participation of key stakeholders. Time and resource constraints also impacted the depth of fieldwork, the number of case studies examined, and the extent of stakeholder engagement, thereby affecting the scope of the study. Moreover, as environmental policies, governance frameworks, and inclusion efforts continue to evolve, some findings may need periodic reassessment to align with emerging trends and developments. Despite these challenges, the study provides critical insights into the systemic barriers that prevent inclusive global environmental governance. It highlights opportunities for policy reforms, capacity-building initiatives, and participatory frameworks that could strengthen representation and engagement. By addressing these limitations in future research, policymakers and stakeholders could develop more effective, inclusive, and sustainable environmental management strategies, ensuring that youth, women, and marginalized communities are meaningfully integrated into decision-making processes.

## Conclusion and Recommendations

This study reveals that many national governments, particularly in less developed regions such as Africa, South Asia, and Latin America, have overlooked the integration of youth, gender, and marginalized perspectives in tackling global environmental challenges. Due to structural and institutional barriers, women, youth, and marginalized groups in these regions remain underrepresented in global decision-making processes. Despite being the majority population and the most vulnerable to environmental risks, their perspectives, needs, and preferences are often overlooked in policies and research, further exacerbating their exclusion from sustainable solutions. The study reveals structural, institutional, and cultural barriers including inadequate funding, absence of youth programs, limited information access, weak capacity-building initiatives, and lack of recognition for women in some developing countries as key drivers of these challenges. Addressing these challenges requires a shift in attitudes, institutional reforms, cultural transformation, educational system improvements, and substantial investments, particularly in developing regions. The study proposes the following recommendations.

1. One of primary findings of the study established that youth, women, and marginalized groups are significantly underrepresented in environmental decision-making processes, limiting the inclusivity and effectiveness of global environmental governance. To strengthen the participation of youth, women, and marginalized groups in global environmental management, leaders must critically assess their roles in promoting inclusive governance. Addressing the limited literature on youth engagement in environmental affairs and actively incorporating diverse perspectives into decision-making is essential. Policies and frameworks should be restructured to ensure equitable representation, capacity-building opportunities, and meaningful participation, fostering more effective and sustainable environmental management strategies.
2. Another important finding of the study revealed that top-down environmental interventions often overlook local expertise and exclude community voices, undermining resilience and long-term sustainability efforts. To avert this, community-led approach integrating indigenous knowledge, participatory research, and grassroots movements is essential for fostering long-term resilience. Leveraging traditional ecological practices for sustainable resource management, this approach would actively engage local populations in data collection and policymaking. Additionally, strengthening advocacy through youth-and women-led environmental initiatives, alongside legal recognition, financial support, and cross-cultural collaboration, would ensures long-term sustainability and equitable environmental governance.
3. The study found that fragmented policies, economic exclusion, and limited access to technology and legal support hinder the active participation of marginalized groups in environmental management. In contrast, this study recommends a multidimensional approach combining policy reforms, economic inclusion, technological empowerment, community-driven strategies, and legal advocacy is crucial for sustainable and inclusive environmental management. This could be achieved by establishing equitable policies, equipping marginalized groups with essential skills and resources, and ensuring financial support for environmental initiatives, fostering long-term resilience and active participation.
4. Another finding indicate that inadequate cross-border collaboration and persistent gender disparities in resource access and decision-making limit equitable participation and hinder progress toward sustainable environmental management. To address this, the study recommend National governments and international organizations should collaborate to foster transboundary dialogues, develop gender-responsive policies, and promote equity by empowering women through resource access, and inclusive decision-making. Strengthening these initiatives would enhance representation and drive sustainable environmental management.
5. The outcome further demonstrate that fragmented efforts, limited stakeholder collaboration, and insufficient support for education and participation are undermining the equity and effectiveness of global environmental management. Addressing this, will require scientific implications of the results highlight the critical need for multifaceted collaborative approach involving governments, NGOs, academia, and local communities to ensure equitable and effective global environmental management. Additionally, the study indicates that enhancing education, financial support, and participatory frameworks could enhance climate resilience, policy effectiveness, and sustainability outcomes.

## Data Availability

No datasets were generated or analysed during the current study.
